# A New Surgical Approach for the Treatment of Conjunctivochalasis: Reduction of the Conjunctival Fold with Bipolar Electrocautery Forceps

**DOI:** 10.1155/2016/6589751

**Published:** 2016-04-21

**Authors:** Eduardo Arenas, Diana Muñoz

**Affiliations:** ^1^Department of Ophthalmology, Colombia National University, Bogota, Colombia; ^2^Department of Physiology, Colombia National University, Bogota, Colombia

## Abstract

*Aim*. To report a new surgical technique for the treatment of conjunctivochalasis.* Methods*. A new surgical technique in which specially designed bipolar electrocautery forceps facilitate the complete reduction of the conjunctival folds without creating lesions near the corneoscleral limbus was designed. A retrospective revision of the medical records of patients treated with this technique between the years 2011 and 2013 was made, and eighteen eyes of sixteen patients with conjunctivochalasis treated with this new technique were included.* Results.* All the eyes treated showed a significant improvement with no evidence of scar lesions after a mean follow-up time of 10 months.* Conclusions*. The surgical technique presented here could be a good alternative for the management of conjunctivochalasis.

## 1. Introduction

Conjunctivochalasis is a condition characterized by the formation of one or several bulbar conjunctival folds on the eyelid margin that alters the lachrymal film physiology in different ways. Its incidence has gradually increased in the population, due to the permanent increase of life expectancy [1, 2]. To manage these folds, different surgical procedures have been described, including the conjunctival fold excision by the use of sutures [3–5], amniotic membrane transplantation [6], and other less invasive procedures using different types of cautery techniques, such as bipolar coagulation [7], laser thermocautery coagulation [8], and argon green laser coagulation [9].

The surgical techniques using excision with sutures, along with other methods that treat the folds close to the limbus, may create scars with the disturbance of the lachrymal film. In this paper we present a new surgical technique in which specially designed bipolar electrocautery forceps facilitate the complete reduction of the conjunctival folds without creating lesions near the corneoscleral limbus.

## 2. Methods

A retrospective revision of clinical records of patients treated with this technique between the years 2011 and 2013 was made. The study adhered to the principles outlined in the Declaration of Helsinki, and verbal and written consent was previously obtained from all patients. Patients who simultaneously underwent other surgical procedures were excluded. Eighteen eyes of sixteen patients, two men and fourteen women, were included. Fold localization, follow-up time, clinical improvement, and the development of complications were evaluated.

### 2.1. Bipolar Electrocautery Forceps

**Figure 1:**
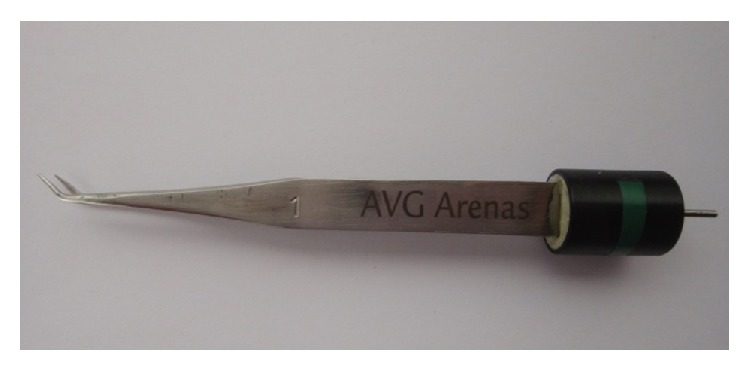
Stainless steel bipolar electrocautery forceps with a curved tip of 3 mm at an angle of 30 degrees.

For this technique, stainless steel bipolar electrocautery forceps were designed with a curved tip of 3 mm at an angle of 30 degrees (Figure 1).

### 2.2. Surgical Technique

This technique begins with the identification of the conjunctival fold with the slit lamp using a lissamine green stain, which will detect where the fold is located and will dye the area responsible for the symptomatology. Then, proparacaine eye drops are instilled into the eye two minutes before placing a 7-0 silk suture at the inferior limbus as a traction point to allow the manipulation of the eye globe and to permit access to the inferior or superior conjunctival fornix as far away as possible from the limbus.

Next, small pieces of Weck cel sponges are placed deep into the lesion site over the span of two minutes. Afterwards, the eyelids are closed with tape, and a topical ocular anesthetic is instilled until the Weck cel sponges have fully absorbed the anesthetic. A three-minute period must be allowed in order to permit a deep penetration of the anesthesia before removing the Weck cel sponges. The surface of the conjunctiva should be dry before performing the next step of the procedure.

**Figure 2:**
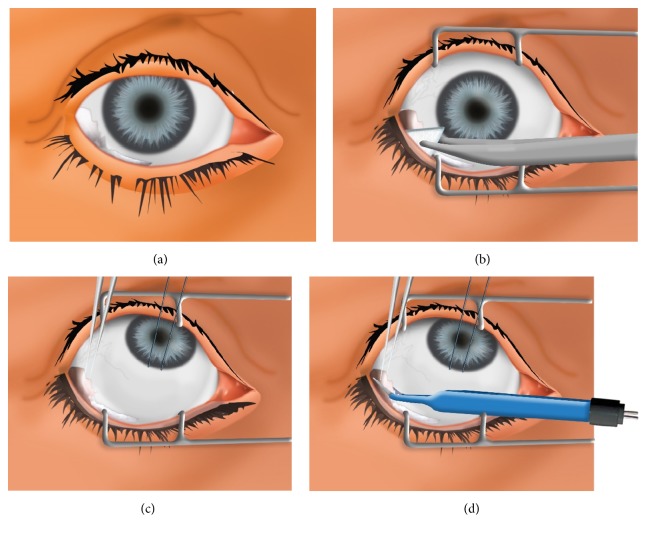
Figure illustrating the surgical technique. (a) Eye globe with temporal conjunctival fold. (b) Small pieces of Weck cel are placed deep into the inferior fornix. (c) The fold is grasped at the center. (d) Heat is applied at the base of the fold with the electrocautery forceps.

A blepharostat is put in place with the aid of dry Weck cel sponges. From now on, while manipulating the cornea, it is important not to irrigate the ocular surface, since it should remain dry in order to identify the exact place where the conjunctival fold is located. Using utility forceps, the conjunctival fold is grasped at the center, as far away from the limbus as possible and close to the fornix. Make sure that the forceps are completely closed. Finally, with the terminal end of the electrocautery forceps slightly wet, heat is applied to the fold with an intensity of 30 mA until complete shrinkage is obtained. The fold should be clamped with the clip as far away as possible from the cornea, so as not to alter the lacrimal film. The procedure should not be repeated on the same fold since just one cauterization is enough to eliminate the redundant tissue. The procedure is repeated on other previously identified folds if necessary (Figure 2).

To finish the surgery, an ophthalmic antibiotic ointment with cortisone is applied and a bandage is put in place. The next day, the bandage is removed and the ointment is continued twice a day for the next two weeks.

## 3. Results

Patient age ranged from 46 to 84 years with a mean age of 64 years. Follow-up time ranged from 1 to 35 months with a mean follow-up time of 10 months. Fourteen eyes corresponded to the right eye and four to the left eye. Twelve eyes had a temporal fold, three a central fold, one a nasal fold, one a superior fold, and one a temporal and inferior fold. At 24 hours postoperatively, all patients showed mild conjunctival hyperemia and edema that resolved in all the cases two weeks later. One patient had moderate ocular pain at 24 hours postoperatively that completely resolved on postoperative day 15. All of the patients showed complete resolution of their symptoms without the development of scar lesions. Finally, none of the patients had any type of ocular or systemic infection.

## 4. Discussion

Conjunctivochalasis is an increasingly common clinical finding in ophthalmic consultation [8, 9], and it is one of the causes of chronic ocular irritation and epiphora, which is sometimes underdiagnosed in the older population. All of the surgical techniques described use surgical excision and placement of sutures, which should be avoided in order to prevent conjunctival scars that sometimes prolong postoperative recovery or create a permanent alteration of the preocular lachrymal film.

**Figure 3:**
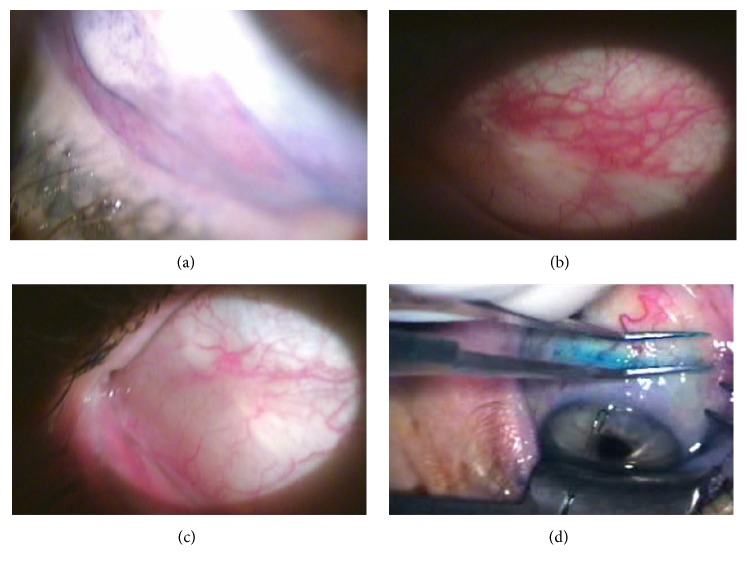
(a) Image showing the temporal conjunctival fold in the right eye of one patient. (b) Postoperative control at 24 hours that shows the complete reduction of the conjunctival fold with no scarring. (c) Postoperative control on day 15 that shows the complete reduction of the conjunctival fold with no scarring. (d) Image showing the application of heat with the electrocautery forceps in another patient.

The technique described here is minimally invasive and painless and shows improvement of the previous signs and symptoms early in the postoperative period, with a total disappearance of the wound at the end of the third week (Figure 3). Additionally, this new technique is very fast, with a mean duration time of 1 minute, and is applicable to the conjunctival folds at any location.
